# Compliance With the Surviving Sepsis Campaign Bundle: A Multicenter Study From Turkey

**DOI:** 10.7759/cureus.14989

**Published:** 2021-05-12

**Authors:** İlhan Bahar, Hafize Oksuz, Nimet Şenoğlu, Hilmi Demirkiran, Mustafa Aydoğan, Yakup Tomak, Mehmet Çömez, Sinem Bayrakçı, Edip Gönüllü, Mustafa Berktaş

**Affiliations:** 1 Anesthesiology and Reanimation, Bakırçay University Çiğli Training and Research Hospital, Izmir, TUR; 2 Anesthesiology and Reanimation, Kahramanmaraş Sütçü imam University Faculty of Medicine, Kahramanmaraş, TUR; 3 Anesthesia and Critical Care, Tepecik Training and Research Hospital, Izmir, TUR; 4 Anesthesiology and Reanimation, University of Van Yuzuncu Yil, Van, TUR; 5 Anesthesia and Critical Care, İnönü University Turgut Özal Medical Center, Malatya, TUR; 6 Anesthesiology and Reanimation, Sakarya University Faculty of Medicine, Sakarya, TUR; 7 Anesthesiology and Reanimation, Mustafa Kemal University Faculty of Medicine, Hatay, TUR; 8 Anesthesiology and Reanimation, Ersin Aslan Training and Research Hospital, Gaziantep, TUR; 9 Anesthesiology and Reanimation, University of Bakırçay, Izmir, TUR; 10 Microbiology, Bakırçay University Faculty of Medicine, Izmir, TUR

**Keywords:** bundle, compliance, turkey, sepsis, guideline

## Abstract

Objectives

Sepsis bundle compliance is not clear. We evaluated rates of compliance with sepsis bundle protocols among health care providers in Turkey.

Methods

Our study was carried out retrospectively. Forty-five intensive care units (ICU) participated in this study between March 2, 2018 and October 1, 2018.

Results

One hundred thirty-eight ICUs were contacted and 45 ICUs agreed to participate. The time taken for the diagnosis of sepsis was less than six hours in 384 (59.8%) patients, while it was more than six hours in 258 (40.2%) patients. The median [interquartile range (IQR)] times for initial antibiotic administration, culturing, vasopressor initiation, and second lactate measurement were 120.0 (60-300) minutes, 24 (12-240) minutes, 40 (20-60) minutes, and 24 (18-24) hours, respectively. The rate of compliance with tissue and organ perfusion follow-up in the first six hours was 0%. The rates of three- and six-hour sepsis bundle protocol compliance were both 0%. The ICU mortality rates for sepsis and septic shock were 22% and 78%, respectively. The ICU mortality rates for sepsis and septic shock were 22% and 78%, respectively.

Conclusions

The rate of compliance with sepsis bundle protocols was evaluated in Turkey for the first time and determined to be 0%.

## Introduction

Sepsis is one of the most common disorders that causes mortality in intensive care units (ICUs) [1]. For the global management of sepsis, international guidelines from the Surviving Sepsis Campaign (SSC) have been published [2]. In 2018, the three- and six-hour bundles were reduced to one hour each by the SSC [3]. Prior observational studies have suggested that the mortality rate and costs of care can be reduced with greater bundle protocol compliance [4,5]. To improve bundle protocol compliance, specific training and infrastructure are necessary [6]; however, according to data from the World Health Organization, the ICUs in 83 countries do not meet minimum standards [7]. The most important reason among those known for ICUs failing to meet standards in this manner is the unavailability of adequate financial support. As compared with developed countries, developing ones typically spend less money and the number of nurses working in these countries is lower [8]; indeed, the number of nurses working in ICUs and the availability of equipment for bronchoscopy, ultrasonography, and hemodynamic monitoring in developing countries are often insufficient to meet patient needs [9]. Moreover, the number of patients per health care professional is high, and the number of beds is limited. Sanitation, electrical resources, ventilation, and patient safety are also often insufficient in hospitals in developing countries [10]. For example, in emergency services, levels of plasma lactate and other biomarkers cannot be measured [11]. Overall, the mortality rate is higher in developing countries [12]. Considering sepsis treatment specifically, according to World Bank data, Turkey is an upper-middle-income country [13]. In developed countries, compatibility with sepsis bundle is high and mortality is low [14]. Sepsis bundle compliance is mandatory in New York [15]. In a previous study conducted in our country, mortality of septic shock was found to be very high [16]. In our country, there is no multi-center study on bundle compliance, which is a mortality-reducing treatment. Our intention was therefore to determine the degree of sepsis bundle protocol compliance in Turkey.

## Materials and methods

Design and setting

Our study was carried out retrospectively. Every eligible medical center was contacted by phone about participating in this study, and the study protocol was sent to them by email. The study protocol is available at https://forms.gle/GRuqdXyAErt2Vmwz5. In total, 138 intensive care units were contacted. Sixty-eight intensive care units were not appropriate. The reason for this is, in 36 intensive care units, the person-in-charge was not defined. Thirty-two intensive care units were specific intensive care units. Seventy intensive care units were eligible for the study. Of these, 35 intensive care units were excluded. The reasons for exclusion were 29 intensive care units did not agree to participate. Six intensive care units provided insufficient data. We worked with 45 intensive care units. In intensive care units, two patients had one nurse and one doctor worked for 24 hours. The study was conducted between March 1, 2018 and October 1, 2018.

Participants

Adult patients older than 18 years were included in this study. All patients with further life expectancy in the ICU were deemed eligible to participate. The inclusion criterion for this study was a diagnosis of sepsis and/or septic shock.

Study data

The prediction or confirmation of infection was performed through 24-hour bedside screening of the study participants. The mortality status of patients discharged from the ICU was determined by reaching the patients or their relatives by phone. The definition of infection was made according to the international sepsis guideline [17]. The presence of sepsis or septic shock was confirmed according to the standards set by the international sepsis datasheet; the definitions of these conditions were produced according to data published in the international sepsis datasheet [2]. The date of admission to the ICU, the infection source, the primary diagnosis, and the comorbidities present were recorded together with the patient’s Acute Physiology and Chronic Health Evaluation (APACHE II) score. The Sequential Organ Failure Assessment (SOFA) score was recorded daily. Data on other comorbidities such as solid organ malignancy, diabetes mellitus, cerebrovascular disorders, chronic obstructive pulmonary disease (COPD), asthma, heart failure (New York Heart Association functional classes I-IV), coronary artery disease, liver failure, chronic renal failure (undergoing dialysis or serum creatinine ≥ 2 mg/dL), immunosuppression (neutropenia < 500 mm^3^), hematologic malignancy, splenectomy, human immunodeficiency virus infection (CD4 < 200 mm^3^), receipt of chemotherapy or radiotherapy in the last six months prior to hospitalization in the ICU, receipt of corticosteroid treatment, and posttransplant status were also recorded.

Definitions of data

Definitions were made according to the Third International Consensus Definitions for Sepsis and Septic Shock (Sepsis-3). Sepsis was considered when an elevation of the Sepsis-related Organ Failure Assessment (qSOFA) score of two or more points occurred outside the ICU or an elevation of the SOFA score of two or more points occurred in the ICU in patients with suspected or proven infection. The qSOFA score is obtained by awarding one point per item when the following are positive: respiratory rate of at least 22 breaths/min, alteration in mental status, Glasgow Coma Scale score of less than 15 points, and systolic blood pressure of 100 mmHg or less. When the SOFA score was two or more points, a preliminary diagnosis of sepsis was made after the exclusion of other probable causes of organ failure and the patient was admitted to the ICU. If the SOFA score manifested an elevation of two or more points, the diagnosis of sepsis was made. Septic shock was defined as a systolic blood pressure of less than 90 mmHg, a mean blood pressure of less than 65 mmHg despite sufficient fluid loading, or a blood lactate level of more than 4 mmol/L (36 mg/dL).

Statistical analysis

Quantitative data were expressed as mean (with 95% confidence interval of mean) and standard deviation from the mean and were compared between groups using Student’s t-Test. Categorical or qualitative data were expressed as frequency with corresponding percentages and were compared between survivors and non-survivors using Chi-square or Fisher’s Exact Test. Level of significance was set at 5% or 0.05 and p < 0.05 was considered significant. Statistical analysis was completed using the Statistical Package for the Social Sciences version 23.0 software program (IBM Corp., Armonk, NY, USA) and Stata software version 15.1 (Stata Corp LLC, College Station, TX, USA).

## Results

**Figure 1:**
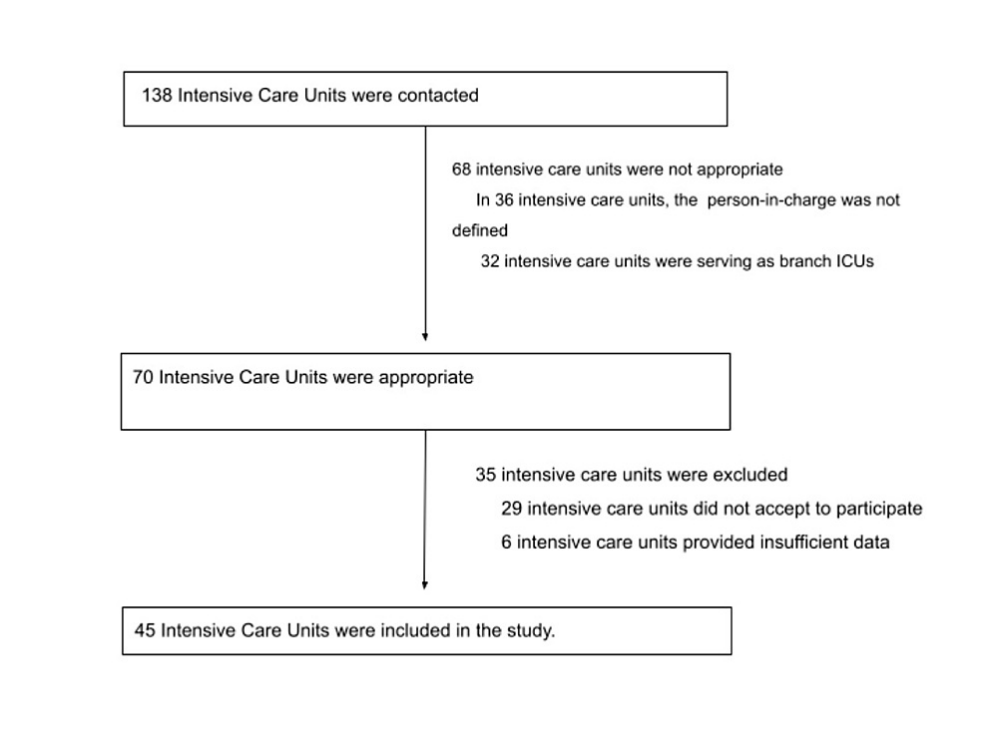
Data collection from intensive care units

A total of 138 ICUs were screened and data were collected from 45 ICUs (Figure 1).

The demographic characteristics of the included patients considering their mortality in view of bundle compliance were similar (Table 1).

**Table 1:** Demographic and clinical characteristics of the patients

Variables	All Patients (N = 642)	Survivors (N = 320)	Non-survivors (N = 322)	P
Patient admission from university hospital, n (%)	172 (27%)	84 (26%)	88 (27%)	0.746
Training and research hospital where patients are admitted, n (%)	316 (49%)	156 (49%)	160 (50%)	
State hospital where patients are admitted, n (%)	154 (24%)	80 (25%)	74 (23%)	
The number of hospital beds, n (%), ≥500	325 (51)	166 (26%)	159 (25%)	0.527
The number of hospital beds, n (%), <500	317 (49)	154 (24%)	163 (25%)	
Age ≥65, years	375 (58%)	189 (29%)	186 (29%)	0.244
Gender Female, n (%)	375 (59%)	189 (29%)	186 (29%)	0.749
BMI (kg/m^2^) 18.5-24.9, n (%)	250 (39%)	119 (37%)	131 (41%)	0.496
BMI (kg/m^2^) 25-29.9, n (%)	120 (19%)	65 (20%)	55 (17%)	
BMI (kg/m^2^) >30, n (%)	272 (42%)	136 (43%)	136 (42%)	
Comorbidity, n (%), COPD	121 (19%)	59 (18%)	62 (19%)	0.212
Comorbidity, n (%), HT	287 (45%)	132 (41%)	155 (48%)	
Comorbidity, n (%), CRF	187 (29%)	102 (32%)	85 (27%)	
Other comorbid diseases, n (%)	47 (7%)	27 (8%)	20 (6%)	
Immunosuppressed patient, n (%)	134 (21%)	48 (15%)	86 (27%)	0.001
Pneumonia as a source of sepsis, n (%)	311 (48%)	145 (45%)	166 (52%)	0.020
Abdominal as a source of sepsis, n (%)	35 (6%)	18 (6%)	17 (5%)	
Urinary tract as a source of sepsis, n (%)	58 (9%)	42 (13%)	16 (5%)	
Bacteremia as a source of sepsis, n (%)	130 (20%)	55 (17%)	75 (23%)	
Sepsis source cannot be identified, n (%)	108 (17%)	60 (19%)	48 (15%)	
Medical intensive care, n (%)	223 (35%)	125 (39%)	98 (30%)	0.793
Surgical intensive care, n (%)	419 (65%)	195 (61%)	224 (70%)	
Number of single antibiotics empirically, n (%)	496 (78%)	261 (81%)	235 (72%)	0.034
Empirically double antibiotic count, n (%)	146 (22%)	59 (19%)	87 (28%)	
APACHE II score <17, n (%)	54 (8.4%)	33 (10%)	21 (6%)	0.084
APACHE II score >17, n (%)	588 (91.6%)	287 (90%)	301 (94%)	
Number of patients taken from the ward to intensive care unit, n (%)	181 (28%)	85 (27%)	96 (30%)	0.584
Number of patients admitted to intensive care from other intensive care, n (%)	159 (25%)	84 (26%)	75 (23%)	
Number of patients admitted to intensive care from the emergency department, n (%)	302 (47%)	151 (47%)	151 (47%)	
Gram-positive microorganism causing sepsis, n (%)	83 (13%)	40 (13%)	43 (13%)	0.584
Gram-negative microorganism causing sepsis, n (%)	285 (44%)	151 (47%)	134 (42%)	
Number of culture negative sepsis, n (%)	130 (20%)	62 (19%	68 (21%)	
Other microorganisms that cause sepsis, n (%)	144 (22%)	67 (21%)	77 (24%)	
Number of septic episodes 1, n (%)	513 (80%)	243 (76%)	270 (84%)	0.012
Number of septic episodes ≥ 2, n (%)	129 (20%)	77 (24%)	52 (14%)	

The three- and six-hour bundle protocol compliance rates were both 0% (Table 2).

**Table 2:** Bundle compliance of patients

Variables	All Patients (N = 642)	Survivors (N = 320)	Non-survivors (N = 322)	P
Septic shock, n (%)	465 (72%)	213 (67%)	252 (78%)	0.001
Sepsis, n (%)	177 (28%)	107 (33%)	70 (22%)	
The time to diagnosing sepsis, n (%) ≤6 hours	384 (60%)	193 (60%)	191 (59%)	0.797
The time to diagnosing sepsis, n (%) >6 hours	258 (40%)	127 (40%)	131 (41%)	
I used bolus fluid 30 ml / kg, n (%)	74 (12%)	40 (13%)	34 (11%)	0.461
I did not use bolus liquid 30 ml / kg, n (%)	568 (88%)	280 (77%)	288 (89%)	
The amount of bolus fluid used (ml), Mean ± SD	1113 ± 676	1148 ± 744	1078 ± 601	0.503
The amount of culture taken in the first hour according to the sepsis bundle recommendation, n (%)	202 (31%)	180 (56%)	22 (6%)	0.001
Cultures were not bundle-compliance in the first hour, n (%)	440 (68.5%)	140 (44%)	300 (94%)	
Antibiotics were administered in the first hour, n (%)	324 (50%)	254 (79%)	70 (22%)	0.001
Antibiotics were not administered in the first hour, n (%)	318 (50%)	66 (21%)	252 (78%)	
First culture time, Median (IQR), min.	24.0 (12.0-240.0)	27.0 (12.0-240.0)	24.0 (12.0-240.0)	0.001
First antibiotic time, Median (IQR), min.	120.0 (60.0-300.0)	90.0 (27.0-120.0)	300.0 (120.0-600.0)	0.001
When was lactate measured first? Median (IQR), min.	110.0 (90.0-150.0)	230 (120-340)	190 (120-340)	0.340
When was the bolus fluid given? Median (IQR), min.	20.0 (12.0-34.0)	23.0 (12.0-34.0)	19.0 (12.0-34.0)	0.529
If MAP < 65 mmHg, when was vasopressor started? Median (IQR), min.	40.0 (20.0-60.0)	34.5 (18.0-59.5)	40.0 (20.0-60.0)	0.069
The time for second lactate measurement, Median (IQR), hours	24.0 (18.0-24.0)	24.0 (26.0-24.0)	24.0 (18.0-24.0)	0.709
One-hour bundle compliance	0 (0%)	0 (0%)	0 (0%)	NS
Three-hour bundle compliance	0 (0%)	0 (0%)	0 (0%)	NS
Six-hour bundle compliance	0 (0%)	0 (0%)	0 (0%)	NS

The 2018 SSC one-hour bundle protocol compliance rate was also 0%. The rate of obtaining an appropriate culture in the first three-hour bundle was 31%, while the rate of using appropriate antibiotics at an appropriate time was 50%. Additionally, the rate of initial administration of an appropriate fluid bolus was 12% and the mean fluid bolus volume was 1,113 ± 676 mL. Further, the initiation of a vasopressor drug if the mean blood pressure was less than 65 mmHg presented a rate of 11.5%, while the rate of second lactate measurement was 0%. The rate of assessing at least two items among the parameters of central venous pressure (CVP) measurement, central venous oxygen saturation (ScvO)2, cardiac ultrasonography, dynamic testing, and passive lower-extremity elevation testing - besides the vital signs, cardiopulmonary findings, capillary refilling, and cutaneous findings - to determine the volume and perfusion status of a patient was 0.3%. The median number of days that a mechanical ventilator was used was 10 (interquartile range: 5-23 days). The mortality rate due to sepsis in the intensive care unit was 22%. The mortality rate due to septic shock in the intensive care unit was 78%.

## Discussion

In our study involving 45 ICUs, we determined that the one-hour bundle compliance rate was 0%. Also, we found that the three- and six-hour bundle compliance rates were each 0%. These results are quite low when compared to those recorded from both developing and developed countries [5,18]. In our study, the most significant deviation from compliance was seen with the absence of recurrent lactate measurements within the one- and six-hour bundles. In a similar study conducted in Asian countries with high- and middle-level incomes, those with low middle and middle-level incomes reported a rate of compliance with the six-hour resuscitation bundle of 6.9% and a mortality rate of 50% [19]. In a study conducted in Bangladesh, which is a low-income country, the six-hour resuscitation bundle compliance rate was 0%, with the hospital mortality rate being 49.2% [20]. In a multicenter study performed in Asia, the team reported a level of 7.6% bundle compliance. After the sepsis training was given to physicians, the bundle compliance rate was 37.5% [18].

A study of developed and developing countries reported a total three-hour bundle compliance rate of 19%. In this study, the highest three-hour bundle compliance was found in North America, with 29%. Mortality is reduced with greater bundle protocol compliance and the highest six-hour compliance is in western Europe, found as 36% [5]. On the contrary, the lowest bundle compliance was found to be 26% in Africa and Central Asia. In large retrospective studies in the United States of America, it has been found that mortality decreases with bundle compliance [21-22].

Multicenter sepsis study was carried out in Turkey. In this study, sepsis bundle compliance is very low and mortality is high [5]. Among the ways to increase sepsis bundle compliance are education (meeting, bedside training, etc.) and increasing the standardization of sepsis treatment (predefined order sets, screening tools, sepsis teams) [23]. Alnababteh et al.'s 24/7 sepsis teams increased the sepsis bundle compliance from 10.7% to 36.9%. They reduced the readmission rate from 5.9% to 4% and the mortality rate from 20% to 16% [24]. Moore et al. used the sepsis screening tool to increase the compliance rate of sepsis bundle and they increased the compliance rate [25]. Schramm et al. established a multidisciplinary response team and increased bundle compliance by 12.7% with weekly feedback [26].

The primary limitation of our study was that it was conducted only in tertiary ICUs and does not represent the conditions of other ICUs or hospital wards. There is therefore a need to conduct clinical trials in developing countries.

## Conclusions

In conclusion, we found bundle compliance very low and mortality high in Turkey. Studies are needed to increase the compliance of sepsis bundle (effect of training, impact of the team, screening and feedback).
